# Improving the delivery of primary care for older people

**DOI:** 10.5694/mja2.50236

**Published:** 2019-06-17

**Authors:** C Dimity Pond, Catherine Regan

**Affiliations:** ^1^ University of Newcastle Newcastle NSW

**Keywords:** Continuity of patient care, Aged, Falls, Health services for the aged, General practice, Health policy, Primary health care, Dementia

*The strengths of primary care should be harnessed to address complexities of the ageing population*

The Australian Institute for Health and Welfare estimates that by 2057 there will be 8.8 million Australians aged 65 years and over, representing 22% of the population. This is an increase from 3.8 million (15% of the population) in 2017.^1^ The Institute also found that although around 70% self‐assess their health as being good, very good or excellent, around 20% overall experience severe or profound core activity limitation. This applies to around 50% by 85 years of age.

In 2017, one‐fifth of all presentations to emergency departments was for people aged 65 years and over,^1^ but multiple inpatient and outpatient hospital attendances are clearly not an effective way to deal with this growing challenge. Primary care providers, with their potential to focus on primary and secondary prevention, their ability to identify disease at an early stage, their knowledge of the patient including their social context and their capacity for ongoing chronic disease management are vital for the health care of this group. Moreover, primary care has been shown to be cost‐effective,^2^ an important consideration in a society where taxpaying workers are a shrinking proportion of the population.

## What is primary care?

Primary care is provided by a range of professionals including but not limited to nursing, allied health and general practitioners, otherwise known as family medicine specialists. In most parts of Australia, GPs provide first contact primary care, although in some places nurses, including nurse practitioners, may be the point of first contact. The World Organization of Family Doctors and the World Health Organization (WHO) describe eight core principles that guide family medicine education and training: access or first contact care; comprehensiveness; continuity of care; coordination; prevention; family orientation; community orientation; and patient centredness.^3^

How do these principles play out in identification and management of disease in older people, including geriatric syndromes, and how should we strengthen this part of the system?

## Access

Primary care practitioners are fairly readily accessible geographically, with the number of full‐time equivalents per 100 000 population ranging from 111.6 in urban areas to 135.5 in remote areas, although remote areas have a range of distance and other geographical barriers to access,^4^ and it may be difficult for people with disabilities to access them. Home visits could strengthen this, but home visits by a person's own GP are less available than they were previously, for multiple reasons,^5^ and these should be addressed. Economic accessibility to primary care is reasonable as many will not charge above Medicare Benefits Schedule (MBS) rebates for pensioners, so it is generally possible for older people to be seen, often by someone they know well (continuity of care), although this is at a cost to the GP. However, presentations are often complex, so care of older people fits less readily into standard (< 20 minutes) MBS consultations. This is evidenced by an upward shift in the last decade in the number of problems managed per consultation, and in the length of consultation.^6^ The MBS is the list of health professional services subsidised by the Australian government. Established in 2015, the MBS Review Taskforce is reviewing the items on the Schedule, including those for general practice (https://www.health.gov.au/internet/main/publishing.nsf/Content/MBSReviewTaskforce). The review report, which is still open for consultation, suggests that longer consultations should be factored into the schedule.

Without an approach to this problem that includes appropriate remuneration, GPs, like other doctors, may respond to incentives. They may diminish a presenting problem in order to spend the limited time completing a better incentivised care plan. Recurrent exacerbations of chronic disease (and acute other illnesses) can mean that routine but important preventive health care is addressed. Home and nursing home visits are not well remunerated. The system should recognise the need for longer consultations for older patients, which generally pay less per minute than do shorter ones, and reward them appropriately.

## Comprehensive and patient‐centred approach

A comprehensive and patient‐centred approach is required to identify geriatric syndromes such as dementia and incontinence. The Royal Australian College of General Practitioners (RACP) does not recommend screening for these conditions, as screening has not been shown to improve outcomes.^7^ The current MBS system, with thought, can be improved by harnessing it to facilitate the comprehensive “case finding” approach recommended by the RACGP guidelines: case finding for a wide range of conditions could be included in the annual health assessment of people aged 75 years and older (75+ health assessment), perhaps moving to screening for conditions in age groups with higher prevalence. A randomised controlled trial of the 75+ health assessment concluded that while there was no demonstrated improvement in overall health status in the assessment group, there was nevertheless an improvement in that group in number of falls, Geriatric Depression Scale score, and self‐rated health.^8^ More recently, a review recommended that the assessment be updated using recent evidence to better assess functional decline.^9^ The British Geriatrics Society Comprehensive Geriatric Assessment Toolkit for Primary Care Practitioners is a useful international model that could be adapted for Australia.^10^ It includes multiple good practice guides, including one for functional assessment. The WHO International Classification of Functioning, Disability and Health framework^11^ might also inform revision of the 75+ health assessment.

Geriatric syndromes often require multidisciplinary management, which is a potential strength of primary care. In Australia, MBS funding for care planning and team care assists coordination but the number of rebatable allied health consultations is often insufficient. Care plans should be patient centred, with consideration of the patient's own goals for care being reviewed regularly, and should include preventive activities, allowing an upskilled practice nurse to address goals around lifestyle issues and self‐management. Given that this ideal is rarely achieved, there is room for more education of patients and all primary care professionals in patient‐centred goal setting with input from family carers, particularly in cases of cognitive impairment. It is important that care plans are understood by all as more than an administrative referral task. Quality improvement activities, longer care plan consultations as suggested by the MBS review, and a tweak towards payment on the basis of follow‐up of the plan and more sustainable practice nurse funding might improve this situation.

## Coordination and continuity of care

An important goal for both patients and funders is to decrease or delay functional decline which causes both decreased quality of life and increased health care costs. Primary care (with continuity of care) is ideally placed to recognise the many risk factors (both early and late) as well as the signs of impending frailty and functional decline. This requires access to and coordination of appropriate and evidence‐based medical and social interventions.

A recent systematic review of continuity of care^12^ demonstrated that it is associated with lower mortality rates, perhaps via some of the mechanisms mentioned above. There is a strong perception that the current health system, which includes many part‐time doctors, discourages continuity,^13^ although part‐timers may actually be preferred by patients in some cases.^14^ An acknowledgement of the importance of continuity through the system (eg, by patient identification of their own GP and potentially voluntary enrolment with a regular GP) might benefit patients and overall health expenditure. The recent revision of funding for GPs visiting residential aged care might encourage patients’ own GPs to continue the relationship.

Care transitions between primary and secondary services are also crucial. Quality care requires that hospitals admit older people promptly if required and for as short a time as possible. It should not be derailed by concerns about bed‐blocking, and step‐down facilities for discharge should be made more available. Good communication with primary care on discharge is essential. The Australian Commission on Quality and Safety in Health Care recently released an important document about pain points in transitions of care, which makes a number of pertinent recommendations.^15^ Consultation participants emphasised the need for discharge planning to be a continuous process. Good discharge planning starts on admission, and contains thorough information about the patient's social and health history and current situation. Consultation participants described situations in which the care team did not identify a patient's health and social needs on discharge. On the other hand, when these needs were identified, there were no arrangements made to meet them.^15^

To maintain patients at home, there should be linkages not only between the primary care team but with social care. This faces many barriers in Australia. My Aged Care (https://www.myagedcare.gov.au/), the Australian Government's central access point for aged care services and information, is clunky and unsuited to older people with poor information technology literacy. It provides some access to social care but does not require health providers to be part of the communication loop. Competing goals may exist; for example, while health providers may be advocating exercise, social care providers may be discouraging walking by provision of a bedside commode. The WHO Guidelines on Integrated Care for Older People^16^ outline some important principles for a more integrated approach between health and social care. At the very least, government initiatives should not foster silos of care.

Medication management requires further attention in older people. They acquire more medications as they acquire more diseases, and subspecialty care is not well equipped to take the broader view. Deprescribing has been shown to be safe and effective when done in a slow and systematic manner.^17^

## Summary

Primary care is well placed for the care of older people. It has been proven to be cost‐effective^2^ and has the potential to relieve health system strain due to the demographic transition. For this to be achieved, policy and practice (including education) should focus on what primary care does well and could do better. This should include consideration of the recent MBS Review, in particular those items that pertain to older people and chronic disease, revision of guidelines for the 75+ health assessment, and care planning and policies that encourage better coordination between multiple primary health and social care services and the hospital system. Any changes should be assessed for factors such as continuity of care discussed above and known to affect the health and wellbeing of older people.

## Competing interests

Dimity Pond has served on the advisory board for Nutricia, and has received funding from a range of primary care organisations for delivery of dementia education.

## Provenance

Commissioned; externally peer reviewed.
